# Field evaluation of a prototype tuberculosis lipoarabinomannan lateral flow assay on HIV-positive and HIV-negative patients

**DOI:** 10.1371/journal.pone.0254156

**Published:** 2021-07-26

**Authors:** John T. Connelly, Alfred Andama, Benjamin D. Grant, Alexey Ball, Sandra Mwebe, Lucy Asege, Martha Nakaye, Brianda Barrios Lopez, Helen V. Hsieh, David Katumba, Job Mukwatamundu, Mayimuna Nalubega, Victoria M. Hunt, Stephen Burkot, Harisha Ramachandraiah, Alok Choudhary, Lech Ignatowicz, Bernhard H. Weigl, Christine Bachman, Jerry Mulondo, Fred Semitala, William Worodria, Abraham Pinter, Beston Hamasur, David Bell, Adithya Cattamanchi, Akos Somoskovi

**Affiliations:** 1 Global Good Fund, Intellectual Ventures, Bellevue, Washington, United States of America; 2 College of Health Sciences, Makerere University, Kampala, Uganda; 3 Infectious Diseases Research Collaboration, Kampala, Uganda; 4 Biopromic AB, Solna, Sweden; 5 Public Health Research Institute Center, New Jersey Medical School, Rutgers University, Newark, New Jersey, United States of America; 6 Makerere University Joint AIDS Program, Kampala, Uganda; 7 Mulago National Referral Hospital, Kampala, Uganda; 8 Karolinska Institute, Solna, Sweden; 9 Division of Pulmonary and Critical Care Medicine, University of California San Francisco (UCSF) and Zuckerberg San Francisco General Hospital, San Francisco, California, United States of America; 10 Curry International Tuberculosis Center, UCSF, Oakland, California, United States of America; The University of Georgia, UNITED STATES

## Abstract

Detection of tuberculosis at the point-of-care (POC) is limited by the low sensitivity of current commercially available tests. We describe a diagnostic accuracy field evaluation of a prototype urine Tuberculosis Lipoarabinomannan Lateral Flow Assay (TB-LAM LFA) in both HIV-positive and HIV-negative patients using fresh samples with sensitivity and specificity as the measures of accuracy. This prototype combines a proprietary concentration system with a sensitive LFA. In a prospective study of 292 patients with suspected pulmonary tuberculosis in Uganda, the clinical sensitivity and specificity was compared against a microbiological reference standard including sputum Xpert MTB/RIF Ultra and solid and liquid culture. TB-LAM LFA had an overall sensitivity of 60% (95%CI 51–69%) and specificity of 80% (95%CI 73–85%). When comparing HIV-positive (N = 86) and HIV-negative (N = 206) patients, there was no significant difference in sensitivity (sensitivity difference 8%, 95%CI -11% to +24%, p = 0.4351) or specificity (specificity difference -9%, 95%CI -24% to +4%, p = 0.2051). Compared to the commercially available Alere Determine TB-LAM Ag test, the TB-LAM LFA prototype had improved sensitivity in both HIV-negative (difference 49%, 95%CI 37% to 59%, p<0.0001) and HIV-positive patients with CD4+ T-cell counts >200cells/μL (difference 59%, 95%CI 32% to 75%, p = 0.0009). This report is the first to show improved performance of a urine TB LAM test for HIV-negative patients in a high TB burden setting. We also offer potential assay refinement solutions that may further improve sensitivity and specificity.

## Introduction

Tuberculosis (TB) remains one of the most vexing infectious diseases, having caused 1.2 million deaths in HIV-negative individuals and an additional 208,000 deaths among HIV-positive individuals in 2019. In the absence of a simple, inexpensive point-of-care screening test to rapidly identify presumptive TB patients for subsequent confirmatory testing, approximately 30% of the annual 10 million new patients with TB remain undiagnosed every year [1].

Lipoarabinomannan (LAM) is a glycolipid of the mycobacterial cell wall that is identifiable in urine of patients with active TB disease [2–5]. LAM offers potential for rapid detection of TB from non-sputum samples using low-cost, instrument-free methods, such as lateral flow assay platforms. One such assay, the Alere Determine™ TB LAM Ag Test (Alere LAM, Abbott Diagnostics, Santa Clara, CA, USA), is currently commercially available. However, the sensitivity and specificity of this assay has been shown to be suboptimal, leading the World Health Organization (WHO) to limit its use to a subset of HIV-infected adults, adolescents, and children (CD4+ T-cell counts ≤200 cells/μL, or seriously ill regardless of CD4+ T-cell count, or have signs and symptoms of TB) [3,6]. The limited use case could hamper adoption in several TB endemic settings.

A test that has utility regardless of HIV status and CD4+ T-cell counts would find fewer barriers to implementation. In particular, a low-cost tool is needed that can facilitate standardized selection of patients for confirmatory testing as part of facility- or community-based active case finding. Identifying these cases before transmission occurs or the disease becomes advanced could significantly change the trajectory of the epidemic. In the present study we report the performance of a prototype TB LAM lateral flow assay (LFA) on fresh urine specimens collected from patients seeking care for TB symptoms in a high TB burden setting.

## Materials and methods

### Study design and population

Between October 2018 and March 2019, we conducted a prospective study of adult (>18 years) patients (both hospitalized and outpatients) being evaluated for pulmonary tuberculosis (TB) at Mulago and Kiruddu Referral Hospitals and Kisenyi Health Center IV in Kampala, Uganda. To select participants, we reviewed the Xpert® testing logs at study health centers daily. On each day, we enrolled consecutive adults with a positive sputum Xpert® MTB/RIF Ultra result, and as many consecutive adults with negative Xpert® MTB/RIF Ultra results, in order to enrich for TB cases. Patients were excluded if 1) Xpert® MTB/RIF Ultra test results were invalid; 2) they were already on anti-TB treatment or had taken agents with anti-TB activity such as amino quinolones in the prior 12 months; or 3) they refused or were unable to provide informed consent. The Makerere University School of Medicine Research and Ethics Committee (No. 2017–020), the Uganda National Council for Science and Technology (No. HS2210), and the University of California San Francisco Committee on Human Research (No. 17–21466) reviewed and approved the original study. The results of this study are reported according to the Standards for Reporting of Diagnostic Accuracy Studies (STARD) guidelines [7].

### Data and specimen collection

Eligible patients submitted two spot sputum samples (A&B) and up to 120mL of spontaneously voided urine. Though sputum induction was available, no patients required induction and all samples were expectorated. Sputum was concentrated for fluorescence smear microscopy (x2) and solid and liquid culture (x2). Urine was analyzed using Multistix 10SG Reagent Strips and Clinitek Analyzer (Siemens Healthcare Diagnostics, Tarrytown, NY, USA), and processed using the prototype concentration method and lateral flow assay (LFA). Patients that self-identified as HIV-positive had CD4+ T-cell counts measured using a BD FACSCalibur Flow Cytometer (BD Biosciences, San Jose, CA, USA). Patients who were unsure or reported negative were tested using the Alere Determine™ HIV Ag/Ab test and confirmed using other diagnostic tests in the algorithm. Patients with discrepant urine TB-LAM LFA and sputum Xpert results, specifically false positives, were followed-up at 1 and 2 months for repeat symptom assessment and sputum-based TB testing.

### Sample preparation for TB-LAM LFA

Freshly collected urine samples were stored on ice packs for no more than 3 hours prior to testing with the prototype LFA. Each urine specimen sample (5mL) was filtered using a 25mm syringe filter (0.45μm) (Whatman, UK) to remove large particles, crystals, and debris. The TB LAM antigen was concentrated from the filtered urine using a concentration system developed at Intellectual Ventures Laboratory. Briefly, the filtered urine was added to the lyophilized Concentration Capture Reagent (the procedure for making this reagent is detailed in the SI), mixed until the reagent was dissolved (approximately 30 seconds), and incubated for 15 minutes at room temperature. The 1μm diameter particles that comprise the Concentration Capture Reagent were separated from the urine using a Whatman Autovial 5mL Syringeless Filter Device (Whatman, UK). The trapped particles were washed with 1mL of Wash Buffer; the urine sample and wash liquid were discarded to waste. The Concentration Capture Reagent particles were treated with 300 μL of Elution Buffer for 5 minutes to release any LAM bound to the particles. The eluted sample was immediately neutralized with 35 μL Neutralization Buffer. The neutralized eluent (150μL) was applied immediately to the LFA.

### Index tests

**Fig 1:**
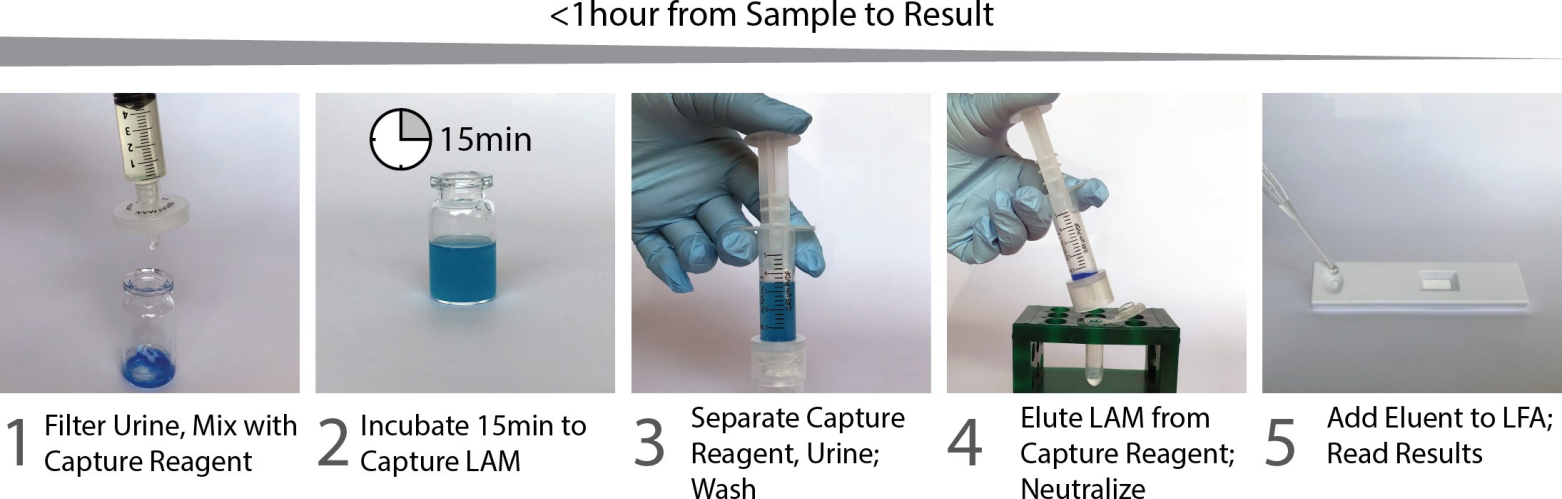
Schematic of the prototype test workflow. (1) Patient urine is passed through a filter to remove any large particulates and mixed with the Concentration Capture Reagent. (2) Urine and Concentration Capture Reagent are allowed to incubate at room temperature for 15min in order to capture any LAM in the sample. (3) The Concentration Capture Reagent is separated from the bulk urine by passing through the filter of an Autovial, discarding the urine, and it is washed with 1mL Wash Buffer, which is also passed through the filter to waste. (4) 300μL of Elution Buffer is added to the washed Concentration Capture Reagent and incubated for 5min to release any captured LAM; it is passed through the filter and instantly mixed with 35μL of Neutralization Buffer. (5) 150μL of this neutralized eluent is added to the LFA and results are read after 30min.

The prototype TB-LAM LFA was custom manufactured by DCN Diagnostics (Carlsbad, California) with a mouse anti-LAM IgG, clone BPM101 (Biopromic AB, Solna, Sweden) as the detection reagent and a human anti-LAM IgM, clone A194-01 (Rutgers University, New Brunswick, NJ) [8] as the capture reagent. The prototype LFA used non-woven materials from Ahlstrom-Munksjö (Helsinki, Finland) and Sartorius (Göttingen, Germany) and the specifications of the build are described in detail in the Supplementary Methods (S1 File). Concentrated TB LAM sample (150μL) was applied to the LFA. The LFAs were scored visually (positive, negative, and invalid) by operators trained by the prototype developers and with an LFA reader, Axxin model AX-2X-DR-G (Axxin, Fairfield, Victoria, Austrialia) at 30 minutes. The combined sample preparation and TB LAM lateral flow assay can be seen in Fig 1. Neat urine was also used to perform the Alere Determine TB-LAM test in accordance with manufacturer recommendations. Assessors of the index tests were blinded to the results of the reference tests and the other index test. Both index tests were performed in near-patient laboratories within either the internal medicine department at Kiruddu Hospital or the TB wards at Mulago Hospital.

### Reference standard

We compared the performance of index tests against a microbiological reference standard consisting of Xpert® MTB/RIF Ultra on sputum and concentrated urine, and liquid and solid cultures on 2 sputum specimens. Urine for Xpert Ultra testing was centrifuged (Thermo Scientific Heraeus Megafuge 8R Compact, Waltham, MA, USA) using swing-out rotor at 3000 rpm (2173 x g) ambient temperature for 15 minutes prior to adding sample reagent at a volume ratio of 1:1. Liquid cultures were performed using the BD BACTEC Mycobacterial Growth Indicator Tubes (MGIT) (BD Biosciences, San Jose, CA, USA) and growth was confirmed as MTB using the SD BIOLINE TB Ag MPT64 Rapid test (Abbott Diagnostics, Santa Clara, CA, USA) on the culture media. Solid cultures were performed using Lowenstein Jensen media. Individual contaminated cultures were counted as negative when compiling the standard. Any missing results from an individual component of the reference standard was ignored as part of the analysis. The microbiological reference standard was considered positive if any one of the tests was positive for TB. Assessors of the reference standard were blinded to index test results and clinical information.

### Statistical analysis

All statistical analysis was performed using R-software version 3.6.0 and the epiR package version 1.0–2 (https://CRAN.R-project.org/package=epiR), when necessary, for example for determination of the sensitivity, specificity, and their associated 95% confidence intervals (95%CI) for the index tests compared to the microbiological reference standard. As the index test is an early prototype device, a sample size was not pre-specified. We continued to enroll patients until the 95% confidence intervals of the sensitivity and specificity for the prototype converged to within ±10%. With these performance characteristics calculated, we again used epiR to compare the sensitivity and specificity of the index tests to each other using McNemar’s test, as the two tests were performed on the same patients in parallel. Here, we considered the TB cases and non-TB cases separately to determine if the sensitivity and specificity, respectively, were significantly different between tests [9], with the null hypothesis–that the two tests were equivalent with respect to the characteristic in question–being rejected when p<0.05. When comparing sensitivity and specificity of the index tests across subsets of patients (e.g. HIV-negative patients versus HIV-positive patients) Fisher’s exact test was employed in the same fashion as described for the McNemar’s test above.

## Results

### Performance of prototype TB LAM test in Uganda

**Fig 2:**
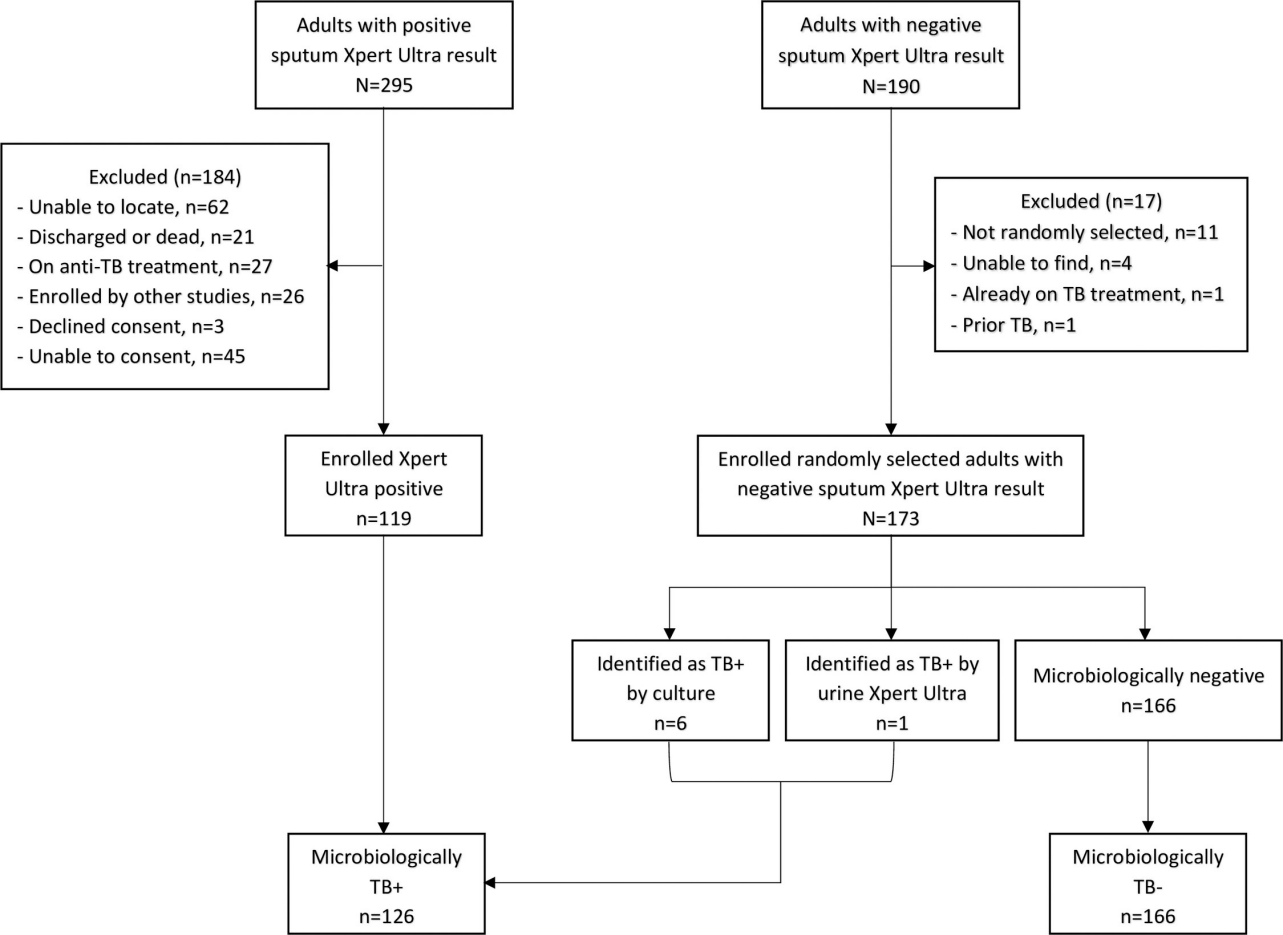
Flowchart of patient enrollment. We identified 295 adults with positive Xpert Ultra results to screen for enrollment. Of these individuals 119 were able to be enrolled in the study. A random selection of 173 adults with negative Xpert Ultra results were also enrolled. Of the 173 enrolled Xpert Ultra negative patients 7 were subsequently diagnosed as TB cases by other microbiological methods.

Between October 2018 and March 2019, we identified 295 patients with positive Xpert results in the facility logbooks. As shown in Fig 2, 119 (40%) were eligible and provided sufficient urine volume for evaluation of the prototype TB-LAM test. We randomly selected 173 patients with negative Xpert results, of these 7 were subsequently confirmed as TB cases based on other microbiological tests, yielding a total of 126 TB+ patients and 166 microbiologically negative patients. Within this group, 206 patients were HIV-negative and 86 were HIV-positive. Amongst the HIV-positive participants, the median CD4+ T-cell count was 262 cells/μL, with 51 having CD4+ T-cell counts above 200 cells/μL, and 67 were taking antiretroviral therapy. Other characteristics of patients enrolled in the study are shown in Table 1.

**Table 1:** Characteristics of participants evaluated for pulmonary TB in Uganda (n = 292).

Demographic or clinical characteristic^a^	All participants, N = 292 (%)
Median Age, years (IQR)	30 (24–39)
Sex	
Female	120 (41)
Male	172 (59)
Cough ≥30 days	186 (64)
Heart rate >120	45 (14)
Fever ≥39°C	6 (2)
BMI <18.5	144 (49)
Hospitalized	19 (6.5)
ECOG Score <2	184 (63)
Smoking in last 30 days	34 (13)
Prior TB (>1 year ago)	39 (13)
HIV-positive	86 (29)
CD4+ T-cell count <200 cells/μL (n = 86)	31 (36)
Reference standard positive	126 (43)
**Distribution of reference positive patients**	
Sputum Xpert Ultra positive (n = 126)	119 (94)
Urine Xpert Ultra positive (n = 126)	18 (14)
MGIT positive (n = 126)	112 (89)
LJ positive (n = 126)	110 (87)

BMI: Body Mass Index; ECOG: Eastern Cooperative Oncology Group; IQR: Interquartile Range.

^a^n = 292 unless indicated.

Based on visual detection of the LFA test line (Table 2), the sensitivity of the prototype TB-LAM LFA was 60% (95%CI 51–69%) and the specificity was 80% (95%CI 73–85%). When restricted to the HIV-negative patients, the sensitivity was 58% (95%CI 47–68%) and the specificity 82% (95%CI 74–89%), not significantly different to the overall population. Similarly, the performance of the test for the HIV-positive patients was not significantly different than the overall cohort–sensitivity was 66% (95%CI 49–80%) and specificity was 73% (95%CI 58–85%). Further stratification of the HIV-positive patients by CD4+ T-cell count indicated that for patients with CD4+ T-cell counts greater than 200, the performance of the prototype is similar to HIV-negative patients–sensitivity is 77% (95%CI 55–92%) and specificity is 82% (95%CI 65–93%). Patients with CD4+ T-cell count less than 200 were not analyzed as a separate subgroup as this group consisted of only 31 patients, a sample size too small to draw significant conclusions from.

**Table 2:** Performance of visually read prototype TB LAM LFA vs. microbiological reference standard.

Cohort	N	TP	FP	FN	TN	Sensitivity [95%CI]	Specificity [95%CI]
**Overall**	292	76	34	50	132	60% [51–69%]	80% [73–85%]
**HIV-**	206	51	21	37	97	58% [47–68%]	82% [74–89%]
**All HIV+**	86	25	13	13	35	66% [49–80%]	73% [58–85%]
**HIV+, CD4+>200**	55	17	6	5	27	77% [55–92%]	82% [65–93%]

Performance shown for the overall cohort and by HIV-status. For HIV+ patients, the performance is also presented stratified by CD4+ T-cell count. TP = true positive; FP = false positive; FN = false negative; TN = true negative; CI = confidence interval.

An attempt to resolve potentially false positive results (n = 34) was made by following up on patients at 1 and 2 months; however, 41% of these individuals (n = 14) were lost to follow-up. Due to this high loss to follow-up, meaningful data could not be collected. This rate of loss to follow-up is not surprising and represents the real-life challenges of conducting evaluations of this nature.

The Alere LAM test was run in parallel with the prototype test, yielding a sensitivity of 13% (95%CI 7–20%) and specificity of 99% (95%CI 96–100%) for the total cohort (Table 3). The low sensitivity of the Alere LAM test in this cohort is due both to the high proportion of HIV-negative patients as well as HIV-positive patients with high CD4+ T-cell counts. This is evidenced by the reduced sensitivity of the test for those patient groups– 9% (95%CI 4–17%) and 18% (95%CI 5–40%), respectively. This is expected as the WHO recommends the test only be used for HIV-positive patients who have signs and symptoms of TB, are seriously ill, or have CD4+ T-cell counts less than 200 cells/μL. The performance of the Alere LAM test within the HIV-positive population of this cohort is in line with previously published reports for outpatient clinic settings and TB symptomatic patients [10,11].

**Table 3:** Performance of the Alere Determine™ TB LAM Ag Test (Alere LAM) vs. microbiological reference standard.

Cohort	N	TP	FP	FN	TN	Sensitivity [95%CI]	Specificity [95%CI]
**Overall**	292	16	2	110	164	13% [7–20%]	99% [96–100%]
**HIV-**	206	8	2	80	116	9% [4–17%]	98% [94–100%]
**All HIV+**	86	8	0	30	48	21% [10–37%]	100% [93–100%]
**HIV+, CD4+>200**	55	4	0	18	33	18% [5–40%]	100% [89–100%]

Performance shown for the overall cohort and by HIV-status. For HIV+ patients, the performance is also presented stratified by CD4+ T-cell count. TP = true positive; FP = false positive; FN = false negative; TN = true negative; CI = confidence interval.

## Discussion

At present 2.9 million of the annual 10 million new tuberculosis cases are estimated to remain undiagnosed annually, this includes 363,574 cases among people living with HIV [1]. In order to address this major diagnostic gap, there is a pressing need for a rapid, simple, inexpensive test that can be easily implemented at a community level where most patients seek initial medical help, to detect likely active tuberculosis cases for further confirmatory testing. To address the diagnostic needs of both HIV-negative and HIV-positive individuals and children as well as patients unable to produce sputum, non-sputum-based approaches are highly desirable.

Against a sputum-based microbiological reference standard, the prototype TB-LAM LFA achieved markedly improved sensitivity compared to the Alere LAM test when prospectively evaluated on specimen collected in a clinical setting. At present the Alere LAM test is recommended for use by the WHO only for a subset of HIV-infected adults, adolescents, and children (CD4+ T-cell counts ≤200 cells/μL, or seriously ill regardless of CD4+ T-cell count, or have signs and symptoms of TB) [6]. The Alere LAM test sensitivity remains best for HIV-positive inpatients, with signs and symptoms of TB, and CD4+ T-cell counts ≤100 cells/μL, 61% (95%CI 40–78%), but significantly lower in patients with higher CD4+ T-cell counts–the pooled sensitivity for patients with CD4+ T-cell counts >200 was found to be 16% (95%CI 8–31%) [6]. The overall sensitivity of the prototype TB LAM LFA was 60% (95%CI 51–69%), with 77% (95%CI 55–92%) in patients with HIV and CD4+ T-cell counts >200 cell/μl, and 58% (95%CI 47–68%) in HIV-negative patients in a cohort where 71.1% of the enrolled patients were HIV-negative and the median CD4+ T-cell count of HIV-positive patients was 262 cell/μl. Therefore, our findings suggest a significant improvement over the Alere LAM test in both HIV-positive patients with higher CD4+ T-cell counts (difference 59%, 95%CI 32% to 75%, p = 0.0009) but more importantly in HIV-negative individuals with tuberculosis (difference 49%, 95%CI 37% to 59%, p<0.0001), two key patient populations that are not adequately covered by the Alere LAM test.

Another recently published TB LAM test reporting improved performance is the Fujifilm SILVAMP TB-LAM (Fuji LAM) test [12]. In contrast to the prototype test reported herein, the Fuji LAM test was initially evaluated only in a retrospective trial on frozen urine samples from HIV-positive patients originally collected for other clinical trials. The median CD4+ T-cell count was 86 cell/μl. The overall sensitivity of the Fuji LAM test was reported as 70.4% (95%CI 53.0–83.1%, n = 968), which is comparable to our 66% (95%CI 49–80%, n = 86) sensitivity on HIV-positive patients. However, the median CD4+ T-cell count in our cohort was significantly higher (262 cells/μl), suggesting a better performance of the prototype in these patients. The additional testing capacity of our test is best illustrated by the striking difference in sensitivity on patients with CD4+ T-cell count >200 cells/μl, 77% (95%CI 55–92%, n = 55) with the prototype and only 44% (95%CI 29.7–58.5%, n = 231) with Fuji LAM.

In a subsequent publication evaluating the performance of the Fuji LAM test in a prospective trail on HIV-negative patients in Peru, the sensitivity compared to a similar microbiological reference was 53.2% (95%CI 43.9–62.1%, n = 372) [13], which is comparable to the sensitivity of our prototype LFA in HIV-negative patients of 58% (95%CI 47–68%). The specificity of Fuji LAM in this HIV-negative was reported as 98.9% (95%CI 96.7–99.6%, n = 372), which is higher than our reported specificity of 82% (95%CI 74–89%). The high specificity of Fuji LAM is credited to the use of an antibody targeting the 5-methylthio-D-xylofuranose (MTX) epitope only found on *Mycobacterium tuberculosis* LAM [12,13].

Based on these findings it appears that this prototype test may open a new path towards a non-sputum-based, rapid and simple LFA testing regardless of the HIV status of the individual. While previous studies using higher sensitivity methods have reported the ability to detect LAM in urine samples from HIV-negative patients, these methods and diagnostic platforms were applicable to reference laboratories due to their complexity [14–16]. Though the Fuji LAM test has now been evaluated in HIV-negative patients, the study did not evaluate its performance in a setting similar to its desired use-case as the samples were centrifuged, the supernatant frozen and shipped to a reference laboratory in Japan [13]. This processing of the urine specimen, necessitated by the need to freeze, presumably is not envisioned as part of the POC use of the Fuji LAM test and may have impacts on the performance. Similarly, a well-resourced reference lab does not replicate the environmental conditions the end-user of the test would experience. Thus, this report is the first clinical evidence from the field on fresh samples in a prospective clinical trial, where the prototype test was performed in a near-patient setting, demonstrating the feasibility of detecting LAM at the POC on an LFA platform. Comparison of the two tests is challenging as one–Fuji LAM–is a final product and our test is a prototype that is under development with future modifications informed by this study. Nonetheless, in their current states both tests take 60 minutes to run and have a similar number of steps [12]. Though our prototype was operable in a POC setting, it certainly requires a reduction in steps to reduce complexity and the potential for user error. This would include eliminating the wash step, integrating neutralization with the LFA via a treated sample pad, and engineering a custom Autovial that would house the lyophilized reagent and allow for both pre-filtration and separation of bound LAM and urine. We envision a final product would contain all the necessary reagents contained within two pieces–the concentration device and the LFA–that would come as a kit.

Critical to the performance of the prototype test is the concentration method. A standard LFA has an upper limit on the specimen volume. By preceding the analysis of the specimen with a concentration method, one enables the sampling of a larger volume–here 5mL–thus making more LAM available for signal generation on the LFA. This approach is likely critical to improving performance in more challenging patient populations (e.g. patients with less advanced disease, HIV- patients and HIV+ patients with high CD4+ T-cell counts) who are thought to generally present with smaller amounts of LAM in urine.

According to the 2014 WHO target product profiles (TPPs) [17] the present specificity of our assay is in line with the minimum and optimal requirements of a non-sputum-based triage test (minimum: ≥90% sensitivity, ≥70% specificity; optimal: ≥95% sensitivity, ≥80% specificity). However, despite the observed increase in sensitivity versus the Alere LAM test on both HIV-negative patients and HIV-positive patients, especially with CD4+ T-cell counts >200 cell/μl, the present performance of the prototype test remains below the recommended minimum sensitivity of the WHO triage test TPP.

In 2018, the UN General Assembly High-Level Meeting on TB targets resolved to close the case detection gap by treating 40 million people with TB by 2022, which requires the additional diagnosis of the 3 million missed cases of tuberculosis annually, on top of what is currently being detected. In order to achieve these goals, we need fundamental changes in current tuberculosis screening practices. Unfortunately, present case finding or screening practices to select presumptive cases for confirmatory testing are based on highly subjective symptom-based screening, which can identify approximately 65% of only the smear positive cases [18], or approaches that require complex and costly infrastructure (e.g. mobile chest X-ray-based screening) [19].

Therefore, in light of this huge diagnostic gap that high disease burden countries are facing, a moderately more sensitive clinic-based screening tool with clear and easily interpretable results, that is inexpensive, simple to use and does not require extensive training or expertise, and that is effective in patients regardless the HIV status or the need to assess that, should have significant clinical and epidemiological impact. This need becomes even more important as wider access to antiretroviral therapy leads to an increasing number of HIV-positive patients with high CD4+ T-cell counts.

In this study, the prototype LFA was validated in a high burden setting, on fresh clinical samples, collected, transported, and tested under real-life circumstances. Although not reaching the requirements of the WHO triage test TPP [17], we believe that with additional optimization based on learnings from this study, further gains in performance are possible. For example, optimizing the concentration method to capture LAM from a larger volume of urine and exploring new antibodies for the LFA, such as an MTX-specific antibody like that used in Fuji LAM, could improve both sensitivity and specificity.

Our evaluation of the prototype had some limitations. All of the patients were capable of producing sputum, limiting our ability to understand how the prototype test may perform in specific populations who have difficulty producing sputum. Also, the number of HIV-positive patients was low, as was the number of patients with positive TB test results in this group, reducing the confidence in the performance of the prototype in this patient group. Another limitation was the lack of microbiological information on potential extrapulmonary disease. Unfortunately, this trial did not allow us to collect extrapulmonary samples for testing. This may have falsely lowered specificity. Furthermore, the sampling performed to recruit Xpert negative patients could have introduced bias. Consequently, we could not calculate positive or negative predictive values. Selection bias is also a concern as the inability to find patients was a common reason for exclusion. Finally, the gold standard of this trial was a composite of molecular and growth detection-based microbiological standards and although we made every attempt to follow up patients with negative microbiological results 1 and 2 months after the initial testing, due to the high rate of loss to follow-up we could not conduct a meaningful discrepant analysis using repeat testing, and control clinical, and radiological evaluation results. This high rate of loss to follow-up is itself an example of why rapid POC testing is necessary. Each contact with a patient is valuable and may be the last time a healthcare worker may have to provide a diagnostic result and link to treatment.

Although the WHO declared tuberculosis a global emergency in 1993, still every day more than 3000 people/day die due to this treatable disease, primarily due to the absence of adequate diagnostic tools at the level where patients are and seek medical help [1]. This report is the first to show an improved TB-LAM LFA performance in HIV-negative individuals on a non-preselected, real-life patient population, which may offer an adequate solution to address the related diagnostic gap. By virtue of its format and relative simplicity, this prototype test demonstrates the possibility to expand TB screening to lower levels of health care systems using urine-based TB LAM testing. Further optimization of the assay and productization of the concentration method may improve performance and the ease of use.

## Supporting information

S1 File Prototype LFA and Concentration Capture Reagent production. (DOCX)

S2 File De-identified patient test results and metadata. (XLSX)

## References

[1] World Health Organization. Global Tuberculosis Report 2020. Geneva; 2020. Available: https://apps.who.int/iris/bitstream/handle/10665/336069/9789240013131-eng.pdf.

[2] Lawn SD. Point-of-care detection of lipoarabinomannan (LAM) in urine for diagnosis of HIV-associated tuberculosis: a state of the art review. BMC Infect Dis. 2012;12: 103. doi: 10.1186/1471-2334-12-103 22536883 PMC3423001

[3] World Health Organization. The use of lateral flow urine lipoarabinomannan assay (LF-LAM) for the diagnosis and screening of active tuberculosis in people living with HIV TB. 2015. Available: https://apps.who.int/iris/bitstream/handle/10665/193633/9789241509633_eng.pdf?sequence=1.

[4] Lawn SD, Dheda K, Kerkhoff AD, Peter JG, Dorman S, Boehme CC, et al. Determine TB-LAM lateral flow urine antigen assay for HIV-associated tuberculosis: recommendations on the design and reporting of clinical studies. BMC Infect Dis. 2013;13: 407. doi: 10.1186/1471-2334-13-407 24004840 PMC3846798

[5] Shah M, Hanrahan C, Wang ZY, Dendukuri N, Lawn SD, Denkinger CM, et al. Lateral flow urine lipoarabinomannan assay for detecting active tuberculosis in HIV-positive adults. Cochrane Database Syst Rev. 2019;2019. doi: 10.1002/14651858.CD011420.pub2 27163343 PMC4916932

[6] World Health Organization. Lateral flow urine lipoarabinomannan assay (LF-LAM) for the diagnosis of active tuberculosis in people living with HIV Policy update (2019). 2019. Available: apps.who.int/iris/bitstream/handle/10665/329479/9789241550604-eng.pdf?sequence=1&isAllowed=y&ua=1.

[7] Bossuyt PM, Reitsma JB, Bruns DE, Gatsonis CA, Glasziou PP, Irwig L, et al. STARD 2015: an updated list of essential items for reporting diagnostic accuracy studies. BMJ. 2015;351: h5527–h5527. doi: 10.1136/bmj.h5527 26511519 PMC4623764

[8] Choudhary A, Patel D, Honnen W, Lai Z, Prattipati RS, Zheng RB, et al. Characterization of the Antigenic Heterogeneity of Lipoarabinomannan, the Major Surface Glycolipid of Mycobacterium tuberculosis, and Complexity of Antibody Specificities toward This Antigen. J Immunol. 2018;200: 3053–3066. doi: 10.4049/jimmunol.1701673 29610143 PMC5911930

[9] Kim S, Lee W. Does McNemar’s test compare the sensitivities and specificities of two diagnostic tests? Stat Methods Med Res. 2014;26: 142–154. doi: 10.1177/0962280214541852 24996898

[10] Nakiyingi L, Moodley VM, Manabe YC, Nicol MP, Holshouser M, Armstrong DT, et al. Diagnostic accuracy of a rapid urine lipoarabinomannan test for tuberculosis in HIV-infected adults. J Acquir Immune Defic Syndr. 2014;66: 270–279. doi: 10.1097/QAI.0000000000000151 24675585 PMC4146703

[11] Peter J, Theron G, Chanda D, Clowes P, Rachow A, Lesosky M, et al. Test characteristics and potential impact of the urine LAM lateral flow assay in HIV-infected outpatients under investigation for TB and able to self-expectorate sputum for diagnostic testing. BMC Infect Dis. 2015; 1–12. doi: 10.1186/s12879-014-0722-x 26156025 PMC4495934

[12] Broger T, Sossen B, Toit E, Kerkhoff AD, Schutz C, Reipold EI, et al. Novel lipoarabinomannan point-of-care tuberculosis test for people with HIV: a diagnostic accuracy study. Lancet Infect Dis. 2019;19: 852–861. doi: 10.1016/S1473-3099(19)30001-5 31155318 PMC6656794

[13] Broger T, Nicol MP, Sigal GB, Gotuzzo E, Zimmer AJ, Surtie S, et al. Diagnostic accuracy of 3 urine lipoarabinomannan tuberculosis assays in HIV-negative outpatients. J Clin Invest. 2020;130: 5756–5764. doi: 10.1172/JCI140461 32692731 PMC7598043

[14] Paris L, Magni R, Zaidi F, Araujo R, Saini N, Harpole M, et al. Urine lipoarabinomannan glycan in HIV-negative patients with pulmonary tuberculosis correlates with disease severity. Sci Transl Med. 2017;9: eaal2807. doi: 10.1126/scitranslmed.aal2807 29237757 PMC6037412

[15] Sigal GB, Pinter A, Lowary TL, Kawasaki M, Li A, Mathew A, et al. A novel sensitive immunoassay targeting the 5-methylthio-D-xylofuranose–lipoarabinomannan epitope meets the WHO’s performance target for tuberculosis diagnosis. J Clin Microbiol. 2018;56: 1–17. doi: 10.1128/JCM.01338-18 30257899 PMC6258851

[16] Wood A, Barizuddin S, Darr CM, Mathai CJ, Ball A, Minch K, et al. Ultrasensitive detection of lipoarabinomannan with plasmonic grating biosensors in clinical samples of HIV negative patients with tuberculosis. PLoS One. 2019;14: 1–12. doi: 10.1371/journal.pone.0214161 30913250 PMC6435140

[17] World Health Organization. High-priority target product profiles for new tuberculosis diagnostics: report of a consensus meeting. 2014. Available: www.who.int/tb.

[18] Baily G V., Savic D, Gothi GD, Naidu VB, Nair SS. Potential yield of pulmonary tuberculosis cases by direct microscopy of sputum in a district of South India. Bull World Health Organ. 1967;37: 875–892. Available: https://www.ncbi.nlm.nih.gov/pubmed/5301646. 5301646 PMC2554240

[19] World Health Organization. WHO Expert Committee on Tuberculosis: ninth report. WHO technical report series no. 552. Geneva: World Health Organization; 1974. Available: https://apps.who.int/iris/handle/10665/41095?show=full. 4216173

