# Supplementary material for: Field evaluation of a prototype tuberculosis lipoarabinomannan lateral flow assay on HIV-positive and HIV-negative patients
Source: PLoS One. 2021 Jul 26;16(7):e0254156. doi: 10.1371/journal.pone.0254156 (PMC8312950; doi:10.1371/journal.pone.0254156)
Supplement: S1 File — (DOCX) [file pone.0254156.s001.docx]

**S1. Supplementary Methods**

**Preparation of Concentration Capture Reagent**

The Concentration Capture Reagent consists of A194-01 IgM antibody conjugated to 1µm carboxylated latex beads (Magsphere Inc., Pasadena, CA) that are lyophilized to provide a room temperature stable reagent. This reagent was prepared in batches and samples of each batch were processed through a quality control protocol to assure consistent performance across batches.

To prepare the beads for conjugation, the stock solution was thoroughly vortexed for 2 minutes to resuspend and then subjected to 40 seconds of alternating 1 second sonic pulses using a probe sonicator set to 25% amplitude in order to disrupt any aggregates. Now a homogenous solution, 400µL of this stock was mixed with 1600µL of 0.1M MES pH6 and again sonicated for 20 seconds with alternating 1 second sonic pulses at 25% amplitude. This suspension was then centrifuged for 5 minutes at 15,000xg and the supernatant was discarded. The beads were then washed by first resuspending them in 950µL of 0.1M MES pH6 by sonication using the settings above and then adding and additional 950µL of 0.1M MES pH6 and vortexing to mix. To wash a second time, the beads were again centrifuged at 15,000xg, discarding the supernatant, resuspending via sonication in 950µL of 0.1M MES pH6, and then adding 762µL of 0.1M MES pH6 and vortexing to mix.

To activate the beads of conjugation, 204µL of freshly prepared 15mg/mL sulfo-NHS in 0.1M MES, pH6 was added to the bead solution and vortexed for 30 seconds. Immediately after vortexing, 34µL of freshly prepared 10mg/mL EDC in 0.1M MES pH6 was added to the bead/NHS solution and again vortexed for 30 seconds and allowed to mix on an orbital shaker for 30 minutes are room temperature. The solution of now activated beads were centrifuged for 5 minutes at 15,000xg and the supernatant discarded. Working quickly, the residual activation reagents were washed out by resuspending the beads in 950µL of 1xPBS with sonication, adding another 950µL of 1xPBS, vortexing, again centrifuging the solution as above, and discarding the supernatant. To conjugate the A194-01 IgM antibody to the beads, the beads were resuspended by sonication in 500µL of 1xPBS and adding 171µL of 2.25mg/mL antibody, vortexing for 30 seconds, and mixing end-over-end overnight at room temperature. To wash away any unconjugated antibody, the total volume was brought to 2mL with 1xPBS and vortexed. The beads were then washed three times by centrifuging to remove the supernatant, sonicating in 1mL 1xPBS as before, and adding another 950µL of 1xPBS and vortexing. The conjugated beads were again mixed end-over-end for 1 hour at room temperature to remove any weakly adsorbed antibody before being subjected to one least wash cycle, as above, resuspending the beads to a final volume of 2mL with 1xPBS.

**Lyophilization of Concentration Capture Reagent**

To provide a shelf-stable reagent that does not require refrigeration, the Concentration Capture Reagent was lyophilized in single use glass vials. A stock solution of lyophilization diluent containing excipients for stabilization was prepared containing 5.31%(w/v) sucrose, 5.31%(w/v) dextran, and 2.66%(w/v) trehalose in 1xPBS. A sufficient volume of this diluent was added to the antibody-conjugated beads prepared above such that the mixture contained a final concentration of 5%(w/v) sucrose, 5%(w/v) dextran, and 2.5%(w/v) trehalose. This diluted mixture was then divided into 800µL aliquots in 5mL glass vials. These vials were then placed in a tray lyophilizer and the tray was brought from room temperature to -52°C at a rate of -1°C/min. The tray was maintained at this temperature under a vacuum of 460 mTorr (instrument settings are in mTorr; 61.33 Pa) for 180 minutes. The tray temperature was raised 0.5°C/min to -31°C and the pressure was dropped to 65 mTorr (8.67 Pa) and held for 999 minutes. The tray temperature was raised 0.2°C/min to -25°C while the pressure was maintained at 65 mTorr (8.67 Pa), once the target temperature was reached these conditions were held for 360 minutes. Maintaining the pressure, the temperature was again raised 0.2°C/min to 30°C and then held under these conditions for 240 minutes. The pressure was then gently increased to 150, 250, 350, and 500 mTorr, each time holding for 10 minutes. Finally, the shelf temperature was reduced to 20°C and the pressure was increased to 900 mTorr (119.99 Pa) until the vials can be removed for immediate capping and sealing.

**Production of prototype LFA**

The prototype LFA, custom manufactured by DCN, consists of 4 non-woven substrates: a glass fiber sample pad (Ahlstrom 8964); a glass fiber conjugate pad (Ahlstrom 8951); a nitrocellulose membrane (Sartorius CN140); and a cotton fiber wick pad (Ahlstrom 320). These materials were treated as described below and assembled onto an 80mm long, 30cm wide, 500µm thick backing card (Lohmann Technologies, Hebron, KY) such that they overlap and create a fluidic path that flows from sample pad, through the conjugate pad, into the nitrocellulose, and, finally into the wick pad as shown in Fig S1. More specifically, the 25mm long nitrocellulose was placed 38mm from the bottom of the backing card, followed by the 29mm long conjugate pad placed 11mm from the bottom card overlapping the nitrocellulose, and the 14mm long sample pad was placed flush to the bottom of the backing card overlapping the conjugate pad. Finally, the 22mm long wick pad was placed 58mm from the bottom of the card, overlapping the nitrocellulose. These cards were then cut into 5mm wide strips, assembled into a plastic cassette, and individually sealed with desiccant in mylar pouches.

14mm 8964 sample pad

25mm CN140 membrane

22mm 320 wick Pad

5mm overlap from 22mm 320 to membrane

3mm overlap between 8964 and 8951

2mm overlap from 29mm 8951 to membrane

80mm Backing Card

29mm 8951 conjugate pad

**Fig S1: Schematic of the Arrangement of Materials on the Prototype LFA**

To prepare the Sartorius CN140 nitrocellulose, the test line was striped 9mm from the bottom edge of the nitrocellulose with 1mg/mL A194-01 IgM, 1%(w/v) sucrose in 1xPBS, pH 7.3 and the control line was striped 14mm from the bottom edge of the nitrocellulose at the same time with 0.5mg/mL goat anti-mouse IgG (Lampire Biological Products, Pipersville, PA), 1%(w/v) sucrose in 1xPBS, pH 7.3 using a contact dispensing system (Biodot, Irvine, CA) at a rate of 2µL/cm. Once dry, the nitrocellulose was blocked with 0.05%(w/v) casein, 0.2%(w/v) sucrose in 5mM borate buffer pH 8, and again dried.

The Ahlstrom 8951 glass fiber conjugate pad was blocked with 0.05%(w/v) casein, 0.2%(w/v) sucrose, in 5mM borate buffer, pH 8 and dried. The signal particles used in the prototype LFA were 400nm diameter carboxylated red latex particles (Magsphere Inc). These were conjugated according to the manufacturer’s instructions with BP101 antibody (Biopromic AB) to yield a 20:1 particle:antibody ratio. These conjugated particles were suspended in 1%(w/v) casein, 10%(w/v) sucrose, 2%(w/v) trehalose in 50mM borate buffer, pH 8 such that the particles were at a final concentration of 0.1%(w/v). This conjugate solution was sprayed onto the blocked pad in two passes, spaced 1cm apart, using a Biodot aerosol dispenser at a rate of 4µL/cm. The Ahlstrom 8964 glass fiber sample pad was treated with 1M Tris, pH9 and dried, while the Ahlstrom 320 wicking pad remained untreated.
